# Dynamic control of spin-wave propagation

**DOI:** 10.1038/s41598-021-86887-8

**Published:** 2021-04-09

**Authors:** Jan-Niklas Toedt, Wolfgang Hansen

**Affiliations:** Institute of Nanostructure and Solid State Physics, University of Hamburg, Luruper Chaussee 149, 22761 Hamburg, Germany

**Keywords:** Ferromagnetism, Spintronics, Electronic and spintronic devices

## Abstract

In this work we present a method to dynamically control the propagation of spin-wave packets. By altering an external magnetic field the refraction of the spin wave at a temporal inhomogeneity is enabled. Since the inhomogeneity is spatially invariant, the spin-wave impulse remains conserved while the frequency is shifted. We demonstrate the stopping and rebound of a traveling Backward-Volume type spin-wave packet.

## Introduction

Spin waves offer an outstanding external controllability of their properties due to the dependence on strength and orientation of externally applied magnetic fields. This appealing possibility is commonly used to alter the system in which a spin wave propagates, e.g., altering the emitted spin-wave patterns of magnetic structures^1,2^, tailoring the band structure in magnonic crystals^3,4^, or tuning the resonance frequency of spin-wave resonators^5–7^. Apart from some exceptions (discussed, e.g. by Chumak et al.^8^), such investigations were limited to statically defined systems, e.g. redirecting spin waves in spatially heterogeneous but statically defined magnetization patterns^9,10^, thus disregarding the capability to dynamically alter the spin-wave properties in the timescale of spin-wave propagation. First investigations on refraction of spin waves at spatial variations of the medium, i.e., a change of the film thickness, were performed most recently^11,12^. In a study by Serga et al. a concept to store spin-wave pulses using a time-variant magnetic field was demonstrated^13^. However, this concept relied on the conversion of propagating spin waves into non-propagating spin-wave modes present in the specific mode scheme of the stripline, limiting the application to a small number of systems. On the other hand, the manipulation of waves by means of a dynamic change of the dispersion relation or the restoring force is an emerging field demonstrated for capillary-gravity waves^14^ and light in waveguides^15^, despite the comparably limited access to a fast controllability of the wave properties. Here, we will expand the concept to the dynamic manipulation of the propagation anisotropy, providing a scheme to remotely and arbitrarily control the propagation of a spin-wave packet. Furthermore, in recent years the generation of isolated Backward-Volume type spin-wave packets has experienced some advances^16,17^. Due to the strong magnetic-field dependence of their group velocity and their highly anisotropic nature, such wave packets are the ideal candidate for the prospect of this article.

In this work we discuss the redirection of running spin-wave packets via refraction at a temporal change of the medium, i.e., by altering an externally applied magnetic field while the spin wave packet propagates. The descriptive boundary condition is the conservation of the wave vector. We demonstrate, by means of micromagnetic simulations, the stopping and rebound of a Backward-Volume type spin wave-packet. We propose the expansion of the concept to remotely steer spin-wave packets by rotating the external magnetic field.

**Figure 1:**
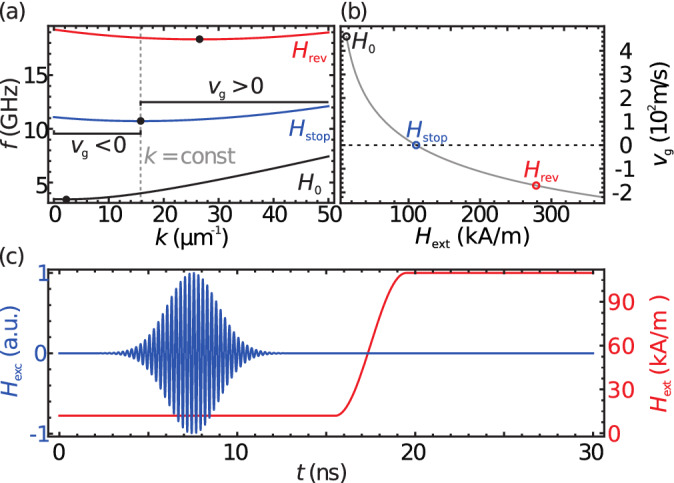
(**a**) Dispersion relations of Backward-Volume waves for three magnetic field strengths. The black dots mark the wave vectors with vanishing group velocity. (**b**) Group velocity at fixed wave number $k=15.8\,\upmu {\hbox {m}}^{-1}$ for varying magnetic field strength. The colored circles mark the field strengths used in panel (**a**). (**c**) Time profile of the exciting magnetic field (blue) and the external magnetic field $H_{{\text {ext}}}$ (red) with target field $H_{{\text {stop}}}={139}\,{\hbox {mT}}$.

The eponymous trait of Backward-Volume waves is their negative group velocity at low wave numbers, meaning that the envelope of a wave-packet and the wave crests travel in opposite direction. All wave numbers above a certain threshold wave number have positive group velocity and a wave number exists at which the group velocity vanishes, i.e. the envelope of a wave packet stands still. Figure 1a displays three dispersion relations at different applied magnetic fields, considering a permalloy film of $t={20}\,{\hbox {nm}}$ thickness, calculated using the model by Kalinikos and Slavin for spin waves in thin films^18^. The black dots mark the wave vectors with vanishing group velocity, which also mark the change from negative to positive group velocity. With rising magnetic field, the threshold wave number increases. If we consider a Backward-Volume wave packet generated at an external field of $\mu _0H_0={16}\,{\hbox {mT}}$ with a central frequency of $f_{{\text {c}}}={4}\,{\hbox {GHz}}$ the central wave number will be $k_{{\text {c}}}={15.8}\,\upmu {\hbox {m}}^{-1}$, according to the dispersion relation. When the magnetic field strength is rapidly altered in a spatially homogeneous fashion, the spatial distribution of the spin-wave packet remains untouched and the wave number is conserved. On the other hand the Zeeman-energy term is changed, thus energy and frequency of the spin wave are altered. As the wave-number distribution of a wave packet is not affected by altering the external field, it is thus possible to shift the threshold wave number onto and beyond the central wave number of the packet, thus effectively stopping or reversing the propagation of a wave packet’s envelope. Figure 1b shows the group velocity of a Backward-Volume wave with the wave number $k_{{\text {c}}}$ in dependence of the external magnetic field strength, calculated as

1 $$\begin{aligned} v_{{\text {g}}}(H_{{\text {ext}}})=2\pi \frac{d}{d k} f(k,H_{{\text {ext}}})\big |_{k=k_{{{\text {c}}}}}, \end{aligned}$$

assuming translation invariance and thus wave-vector conservation. If spatial heterogeneities of the medium or magnetic field occur (e.g. at the borders of a magnetic film) the wave-vector composition of the wave packet will be altered.

## Methods

A set of micromagnetic simulations, using OOMMF^19^, was conducted to verify this concept. Here we consider a $20\,{\hbox {nm}}$ thick permalloy film, which is $320\,\upmu {\hbox {m}}$ long in the Backward-Volume direction (hereafter *x* direction). In the out-of-plane direction (here after *z* direction) the simulation volume is constrained to a single cell. In *y* direction the simulation volume extents over 4 cells ($20\,{\hbox {nm}}$) and translation symmetry is approximated by periodic boundary conditions. The exchange constant used is $A=1.3 \times 10^{-11}\,{\hbox {J m}}^{-3}$; the saturation magnetization was set as $M_{{{\text {S}}}}={803.7}\,{\hbox {kA m}}^{-1}$. Our material of choice for purpose of demonstration is permalloy to stress that the demonstrated approach does not depend on the choice of the specific material. To enhance the quality of the simulations we chose $\alpha =2\times 10^{-3}$ as Gilbert damping parameter, a value which is only a quarter of the real Gilbert damping parameter in permalloy. It is still achievable using other materials, such as Cobalt alloys^20^.

**Figure 2:**
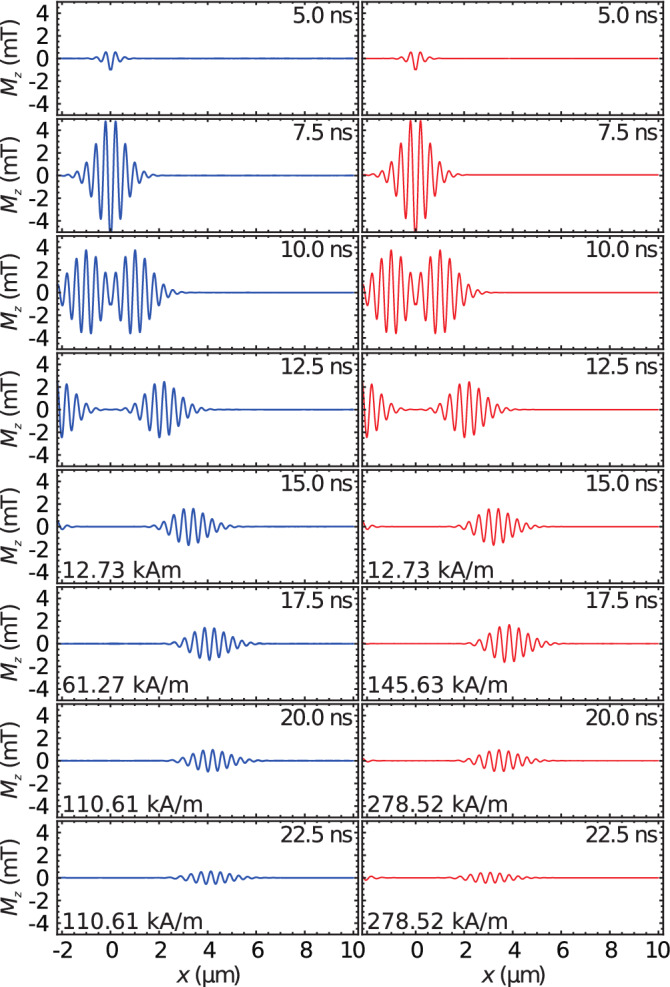
Plots of $M_z$ for the simulations of a stopped (left-hand side) and a reversed (right-hand side) Backward Volume pulse.

A modulated Gaussian pulse of the out of plane magnetic field ($H_z$) with a modulation frequency of $f_{{{\text {c}}}}=4\,{\hbox {GHz}}$ and FWHM of ${3.3}\,{\hbox {ns}}$ excites a Backward-Volume pulse with a central wave vector of $k_{{{\text {c}}}}={15.8}\,{\upmu {\hbox {m}}}^{-1}$. The spatial distribution of the excitation field is of Gaussian shape with a FWHM of ${133}\,{\hbox {nm}}$ in the center of the film ($x=0$), exciting two wave packets propagating in opposite directions. For the example of stopping a wave packet, the magnetic field is increased from $\mu _0H_0={16}\,{\hbox {mT}}$ to $\mu _0H_{{\text {stop}}}={139}\,{\hbox {mT}}$ over ${4}\,{\hbox {ns}}$, beginning ${8}\,{\hbox {ns}}$ after the pulse peak. The time dependences of the exciting magnetic field pulse and the external magnetic field are depicted in Fig. 1c. For the demonstration of propagation reversal, the target magnetic field strength is substituted with $\mu _0H_{{\text {rev}}}={350}\,{\hbox {mT}}$.

Due to the finite size of the simulated film in the direction of the magnetic field (*x*) an in-plane demagnetization field remains, weakening the total internal field. The demagnetization field is calculated following Joseph and Schlömann^21^, retaining a value of $\mu _0H_{{\text {demag}}}={1.0}\,{\hbox {mT}}$ at $x=0$. A corrected form of the dispersion relation for thin films by Kalinikos and Slavin^18^ was obtained by subtracting the demagnetization field from the externally applied field, yielding

2 $$\begin{aligned} f=\frac{\gamma \mu _0}{2\pi }\sqrt{\left( H_{{\mathrm {ext}}}-H_{{\text {demag}}}+\frac{2A}{\mu _0 M_{{\mathrm {S}}}} \right) \cdot \left( H_{{\mathrm {ext}}}-H_{{\text {demag}}} + \frac{2A}{\mu _0 M_{{\mathrm {S}}}}k^2+M_{{\mathrm {S}}}F \right) }, \end{aligned}$$

where $H_{{\mathrm {ext}}}-H_{{\text {demag}}}$ is the static internal field, with the gyroscopic ratio $\gamma ={176}\,{\hbox {GHz T}}^{-1}$, the film thickness *t* and the vacuum permeability $\mu _0={1.625}\,{\upmu {\hbox {H m}}}^{-1}$. The in-plane wave-vectors parallel and orthogonal to the static magnetic field are denoted by $k_{||}$ and $k_{\bot }$, respectively. Furthermore, *F* is given by

3 $$\begin{aligned} F=1-P\frac{k_{||}^2}{k^2}+P\left( 1-P\right) \left( \frac{M_{{\mathrm {S}}}}{H_{{\mathrm {ext}}}-H_{{\text {demag}}}+\frac{2A}{\mu _0M_{{\mathrm {S}}}}k^2} \right) \frac{k_{\bot }^2}{k^2}, \end{aligned}$$

with

4 $$\begin{aligned} P=1-\frac{1-e^{-k t}}{k t}, \end{aligned}$$

Here we neglect dynamic in-plane demagnetization fields arising due to the finite lateral extent of the ferromagnetic film, since the static correction is sufficient for the purpose of this paper.

## Results

**Figure 3:**
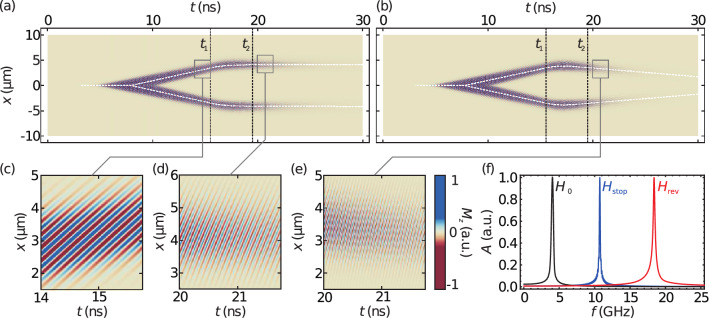
(**a**,**b**) Plots of the out-of-plane magnetization $M_z$ for simulations with an increase of the external magnetic field from $\mu _0H_0={16}\,{\hbox {mT}}$ to $\mu _0H_{{\text {stop}}}={139}\,{\hbox {mT}}$ (**a**) and $\mu _0H_{{\text {rev}}}={350}\,{\hbox {mT}}$ (**b**). (**c**–**e**) Closeups of of $M_z$ (**f**) Plots of the normalized temporal Fourier transforms at $H_0$ (black), $H_{{\text {stop}}}$ (blue), and $H_{{\text {rev}}}$ (red).

An excerpt of the simulation is depicted in Fig. 2. In the left hand panel the increase of the external field to $\mu _0H_{{\text {stop}}}={139}\,{\hbox {mT}}$ is plotted, while the right hand panel shows the simulation for an increase to $\mu _0H_{{\text {rev}}}={350}\,{\hbox {mT}}$. The spin-wave packets are formed at $x=0$, propagating in both directions. The packets separate from another and continue to propagate with constant velocity. After the field increase (beginning at $t={15.5}\,{\hbox {ns}}$) the envelope of the wave packet remains fixed for the left hand panel, while the direction of propagation is reversed for the right hand panel.

Figure 3a,b show the Magnetization component $M_z$ plotted against the coordinate *x* and the elapsed time *t* for both the stopping as well as the reversal of the spin wave packet. The white dashed lines indicate the maxima of the spin-wave pulses; the black dashed lines mark the beginning $(t_1)$ and end $(t_2)$ of the field increase. By tracing the position of the wave envelope’s maxima the central group velocity can be determined. The unaltered wave packet exhibits a group velocity of $v_0={450.22}\,{\hbox {m s}}^{-1}$. At $H_{{\text {stop}}}$ the packet exhibits a residual group velocity of $v_{{\text {stop}}}={-0.88}\,{\hbox {m s}}^{-1}$, and the wave packet at $H_{{\text {rev}}}$ has a group velocity of $v_{{\text {rev}}}={-171.07}\,{\hbox {m s}}^{-1}$, hence propagating back towards the origin of the wave packet. Closeups of the vertical Magnetization $M_z$ are provided in Fig. 3c–e.

By taking the Fourier transform of the magnetization using time windows before and after the field increase, the frequency distributions for the respective windows can be determined as depicted in Fig. 3f. Due to different window lengths available for the Fourier transform, the widths of the spectral distributions are not comparable. The initial central frequency of the pulse is, in agreement with the excitation, ${4.02}\,{\hbox {GHz}}$. At $H_{{\text {stop}}}$ and $H_{{\text {rev}}}$ the central frequency increases to $f_{{\text {stop}}}={10.76}\,{\hbox {GHz}}$ and $f_{{\text {rev}}}={18.42}\,{\hbox {GHz}}$, respectively. The expected frequencies, according to the dispersion relation, are $f_{{\text {stop}}}={10.72}\,{\hbox {GHz}}$ and $f_{{\text {rev}}}={18.51}\,{\hbox {GHz}}$.

It is noteworthy that the spin-wave packets decay quicker after the field increase, especially for $H_{{\text {rev}}}$. The cause for the accelerated attenuation is found in the frequency dependence of the magnon lifetime^22,23^. Since the lifetime decreases with increasing spin-wave frequency, and the frequency of a spin wave with fixed wave number increases with the magnetic field, the lifetime is reduced when the magnetic field grows.

**Figure 4:**
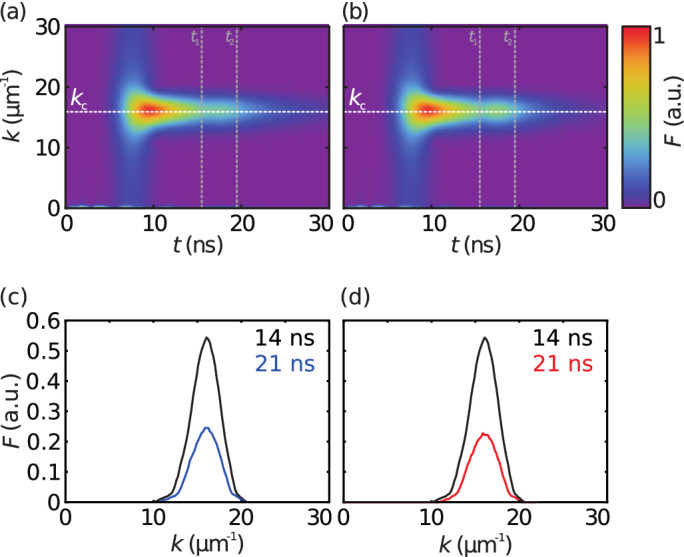
Time dependent spatial Fourier transform of $M_z$ for simulations of a field increase to $H_{{\text {stop}}}$ (**a**) and $H_{{\text {rev}}}$ (**b**). The white dashed line marks the central wave number $k_{{\text {c}}}$ determined from the Fourier transforms. Profiles of the spectra at $t={14}\,{\hbox {ns}}$ and $t={21}\,{\hbox {ns}}$ are provided in panels (**c**) and (**d**) for simulations of a field increase to $H_{{\text {stop}}}$ and $H_{{\text {rev}}}$, respectively.

The wave-number distribution of the spin waves is depicted in Fig. 4. The excited central wave number is determined from the spatial Fourier transform as $k_{{\text {c}}}={15.8}\,{\upmu {\hbox {m}}}^{-1}$, which is also the intended central wave number. Most importantly, in both simulations, the central wave number remains unaltered during and after the field increase in agreement with the postulated conservation of the wave number. In Fig. 4c,d profiles of the wavevector spectra are depicted for times $t={14}\,{\hbox {ns}}$ just before and $t={21}\,{\hbox {ns}}$ after the field increase.

Additionally, in Fig. 4b a boost of the Fourier amplitude is discernible during the field increase, attributed to the raising of the ratio between the external bias field and the dynamic out-of-plane demagnetization field. Since the out-of-plane demagnetization field reduces the out-of-plane component $M_z$ of the spin-wave, and thus the relative strength of the dynamic out-of-plane demagnetization field, the out-of-plane spin-wave amplitude increases with the external magnetic field.

## Extension to redirection of spin waves

**Figure 5:**
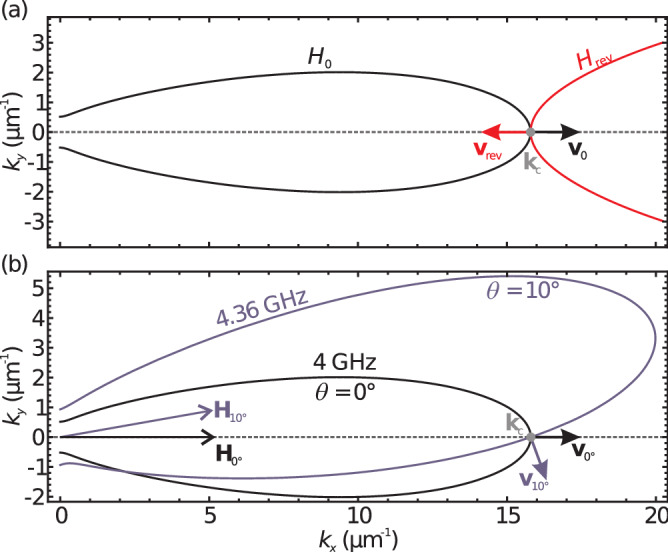
(**a**) Illustration of the reversal of a spin-wave packet in the reciprocal space. The initial isofrequency curve, belonging to $f_0={4}\,{\hbox {GHz}}$ and an external magnetic field of $\mu _0H_0={16}\,{\hbox {m T}}$ is marked in black. After an increase of the magnetic field to $\mu _0H_{{\text {rev}}}={350}\,{\hbox {m T}}$ the central frequency shifts to $f_{{\text {rev}}}={18.51}\,{\hbox {GHz}}$, according to the wave-vector conservation. The resulting isofrequency curve is depicted in red. Initial and resulting group velocity are denoted as black and red arrows, respectively. (**b**) Isofrequency curves for the case of a magnetic-field tilt of $10^{\circ }$. The initial isofrequency curve is depicted in black. Marked in blue is the isofrequency curve for the tilted external field. Again, due to wave-vector conservation the central frequency adapts to $f_c={4.16}\,{\hbox {GHz}}$. The initial and resulting group velocities are denoted as black and blue arrows, respectively.

Based on the demonstrated wave-number conservation the concept of the dynamic propagation control can be extended to the two-dimensional case. For demonstration purposes we will again discuss a BV wave packet with a central frequency of $f_{{\text {c}}}={4}\,{\hbox {GHz}}$ and the same medium as before. Here it is helpful to use the concept of the isofrequency curve, which is the set of wave vectors with identical frequency, according to the dispersion relation. The group velocity, which is the gradient of the frequency in *k* space, is always perpendicular on the isofrequency curve. In Fig. 5a reversal of the wave-packet propagation is depicted. The black line marks the isofrequency curve for the wave packet’s initial central frequency of $f_{{\text {c}}} ={4}\,{\hbox {GHz}}$ at an external field of $\mu _0 H_{0}={16}\,{\hbox {m T}}$, while the red curve marks the isofrequency curve at an external magnetic field of $\mu _0H_{{\text {rev}}}={350}\,{\hbox {m T}}$. Both isofrequency curves encompass the central wave vector $k_c$ (gray dot) as required by the wave-vector conservation. The initial group velocity ${{\mathbf {v}}}_0$ is marked by a black arrow and group velocity ${{\mathbf {v}}}_{{\text {rev}}}$ after the field increase is marked by a red arrow. Figure 5b illustrates a rotation of the external magnetic field. Again, the black line depicts the isofrequency curve for the initial situation, i.e., the field is oriented in *x* direction ($\theta =0$). All parameters are identical to the precedent simulations. If, for example, the external field is tilted by $10^{\circ }$, the static orientation of the magnetization adapts accordingly and the long axis of the isofrequency curve is also tilted by $10^{\circ }$. Considering a spatially homogeneous field-orientation change, the wave vector, again, remains conserved and the frequency of the spin-wave packet is altered. This means, the isofrequency curve for the new central frequency and field orientation will still include the unaltered central wave vector, as marked by the grey dot. The central frequency changes accordingly to $f_{{{\text {c}}}}={4.36}\,{\hbox {GHz}}$. The according isofrequency curve is depicted in Fig. 5 in blue color. The group velocity ${{\mathbf {v}}}_{10{^{\circ }}}$ belonging to $k_{{{\text {c}}}}$ is now tilted by ${70}^{\circ }$. It is thus possible to remotely steer spin-wave packets by rotating an external magnetic field. In combination with altering the field strength any orientation of the group velocity is achievable. Furthermore a Backward-Volume spin wave can be converted to a Damon–Eshbach spin wave (strongly boosting the frequency) and vice versa by rotating the external field ${90}^{\circ }$.

## Conclusion

In summary we propose a method to steer spin-wave packets utilizing dynamic alterations of an external magnetic field. Micromagnetic simulations validate the introduced concept for the propagation control of Backward-Volume wave packets. By choosing the target magnetic field the resulting group velocity of the wave packet can be chosen. It is possible to stop or even reverse the packet propagation. The results of the simulations are in good agreement with the model, especially the wave number remains conserved. The method is expandable to the two-dimensional case, where spin-wave packets can be remotely steered via orientation and strength of the external field. Due to recent advances both in the generation of suitable Backward-Volume packets^24,25^, and the change of the magnetic field in the timescale of few nanoseconds^26^, the proposed concept appears feasible although still challenging.
